# Oral Supplementation with AHCC^®^, a Standardized Extract of Cultured *Lentinula edodes* Mycelia, Enhances Host Resistance against SARS-CoV-2 Infection

**DOI:** 10.3390/pathogens12040554

**Published:** 2023-04-03

**Authors:** Ankita Singh, Awadalkareem Adam, Leslie Rodriguez, Bi-Hung Peng, Binbin Wang, Xuping Xie, Pei-Yong Shi, Kohei Homma, Tian Wang

**Affiliations:** 1 Department of Microbiology & Immunology, University of Texas Medical Branch, Galveston, TX 77555, USA; 2 Department of Neuroscience, Cell Biology and Anatomy, University of Texas Medical Branch, Galveston, TX 77555, USA; 3 Department of Biochemistry & Molecular Biology, University of Texas Medical Branch, Galveston, TX 77555, USA; 4 Sealy Institute for Vaccine Sciences, University of Texas Medical Branch, Galveston, TX 77555, USA; 5 Institute for Human Infections and Immunity, University of Texas Medical Branch, Galveston, TX 77555, USA; 6 Research and Development Division, Amino Up Co., Ltd., Sapporo 004-0839, Hokkaido, Japan; 7 Department of Pathology, University of Texas Medical Branch, Galveston, TX 77555, USA

**Keywords:** nutrient supplementation, SARS-CoV-2, host immunity, treatment, COVID-19

## Abstract

The coronavirus disease 2019 (COVID-19) pandemic has significantly impacted global public health safety and the economy. Multiple antiviral drugs have been developed, and some have received regulatory approval and/or authorization. The use of nutraceuticals can be beneficial for preventing and treating COVID-19 complications. AHCC is a standardized, cultured extract of an edible mushroom *Lentinula edodes* of the *Basidiomycete* family of fungi that is enriched in acylated α-1,4-glucans. Here, we evaluated the effects of the oral administration of AHCC on the host response to SARS-CoV-2 infection in two murine models, K18-hACE2 transgenic mice and immunocompetent BALB/c mice. Oral administration of AHCC every other day for one week before and one day post SARS-CoV-2 infection in both strains of mice decreased the viral load and attenuated inflammation in the lungs. AHCC treatment also significantly reduced SARS-CoV-2-induced lethality in the K18-hACE2 mice. AHCC administration enhanced the expansion of γδ T cells in the spleen and lungs before and after viral infection and promoted T helper 1-prone mucosal and systemic T cell responses in both models. In AHCC-fed BALB/c mice, SARS-CoV-2 specific IgG responses were also enhanced. In summary, AHCC supplementation enhances host resistance against mild and severe COVID-19 infection primarily via the promotion of innate and adaptive T cell immune responses in mice.

## 1. Introduction

The current coronavirus disease 2019 (COVID-19) pandemic has had a significant global impact on public health safety and socioeconomic activities over the past two and half years. While the majority of COVID-19 cases are mild or asymptomatic, 5 to 14% of COVID-19 patients develop severe pneumonia or critical multiorgan failure or die [1,2]. Extrapulmonary manifestations, including neurological symptoms, ranging from mild specific or nonspecific symptoms to life-threatening encephalopathy and central nervous system (CNS)-mediated respiratory stress have also been reported [3,4]. Global efforts have been focused on the development of methods to prevent and treat COVID-19 diseases. Four vaccines and a number of antiviral treatments have received emergency use authorization (EUA) and/or regulatory approval around the world [5,6]. Despite the development of successful measures for prevention and treatment, continuous efforts to develop alternative solutions to decrease or attenuate COVID-19 related complications are needed.

Acute SARS-CoV-2 infection has been studied in various animal models, including hamsters, ferrets, nonhuman primate (NHP)s, rats, and mice [7,8]. Among them, infection in hamsters and ferrets partially recapitulates clinical symptoms in humans, although there are limited reagents available to study infection and pathogenesis in these models. Unlike NHPs, mice are relatively low in cost, easy to work with, and are most amenable to immunological manipulation. Angiotensin converting enzyme 2 (ACE2) is the cell entry receptor for SARS-CoV-2 [9]. Mouse ACE2 shows key differences from human ACE2 and is generally resistant to SARS-CoV-2 infection. However, SARS-CoV-2 replicates in the lung tissues of the K18-hACE2 transgenic mice display weight loss and interstitial pneumonitis, similar to what has been described in humans. Encephalitis-related lethality was also induced in these mice due to virus dissemination into the brain [10,11]. Moreover, the delivery of adeno-associated virus (AAV)–mediated expression of hACE2 into the respiratory tracts of immunocompetent mice causes more productive infections in these mice with mild acute respiratory distress syndrome [12]. The mouse-adapted SARS-CoV-2 (SARS-CoV-2 MA or CMA) strain, which incorporates key mutations that allow the viruses to use mouse ACE2 for entry into cells, also infects the lung and causes mild clinical diseases in young adult immunocompetent mice [13,14]. 

AHCC, the trademark of Amino Up Co., Ltd., is a standardized extract of cultured *Lentinula edodes* mycelia which contains oligosaccharides, amino acids, lipids, and minerals. The main component of AHCC is *α*-1,4-glucans, and the glucan fractions have been associated with the biological activities of AHCC [15]. Previous studies have documented the antitumor effects of AHCC supplementation in animal models [16,17]. In addition, in vitro and in vivo animal studies suggest that AHCC increases host defense against microbial pathogens, including bacterial, fungal, and viral infections, via the modulation of the functions of immune cells [18,19,20]. In the present study, we evaluated the effects of oral supplementation with AHCC on SARS-CoV-2 infection in K18-hACE2 mice and immunocompetent BALB/c mice displaying severe and mild clinical symptoms, respectively. In both mouse models, we found that supplementation with AHCC prior to and after infection with SARS-CoV-2 decreased viral replication and virus-induced inflammation and pathology in the lung as well as decreasing lethality in K18-hACE2 mice.

## 2. Materials and Methods

### 2.1. Viruses

The SARS-CoV-2 USA-WA1/2020 strain was obtained from the World Reference Center for Emerging Viruses and Arboviruses (WRCEVA) at the University of Texas Medical Branch (UTMB) and was amplified twice in Vero E6 cells. The mouse-adapted SARS-CoV-2 CMA4 strain was generated as described previously [14]. 

### 2.2. Mice

K18-hACE2 mice (Jackson Lab stock #034860, Bar Harbor, MME, USA) were bred and maintained at the UTMB animal facility. BALB/c mice were purchased from the Jackson Laboratory.

### 2.3. SARS-CoV-2 Infection in Mice

Six-week-old mice were infected intranasally (i.n.) with 1 × 10^4^ plaque-forming units (PFU) of the SARS-CoV-2 mouse-adapted CMA4 strain or 5 × 10^3^ PFU of the SARS-CoV-2 USA-WA1/2020 strain. Infected mice were monitored twice daily for morbidity and mortality. In some experiments, on days 2, 4, 6, and 8 post infection, mice were euthanized for blood and tissue collection to study the viral load, lung pathology, sera antibody titers, and immune cell functions.

### 2.4. Oral Feeding with AHCC

AHCC (Amino Up Co., Ltd., Sapporo, Japan) was dissolved in distilled water. Mice were orally administered with AHCC (360 mg/kg as FD; freeze-dried powder) by gavage every other day for 8 days at a Biosafety level (BSL) 2 animal facility before being transferred to a BSL3 animal facility for infection and fed at day 1 post-infection in a 100 μL volume. Control mice received 100 μL of distilled water. Noninfected samples were collected following feeding five times with water or AHCC. Based on body weight, similar doses of AHCC were used in a previous study and showed no toxic effect in mice [21,22].

### 2.5. Quantitative PCR (Q-PCR)

The sequences of the primer sets (IDT Technologies, Coralville, IA, USA) for cytokines, chemokines, SARS-CoV-2 spike (S) gene, β-actin, and PCR reaction conditions were described previously [23,24,25,26]. Lung and brain tissues were resuspended in TRIzol for RNA extraction in accordance with the manufacturer’s instructions (Life Technologies, Carlsbad, CA, USA). The RNA concentration and purity were determined using a WPA Biowave DNA Spectrophotometer. Complementary (c) DNA was then synthesized with a qScript cDNA synthesis kit (Bio-Rad). Gene expression levels of SARS-CoV-2 S and mouse inflammatory cytokines and chemokines (IL-1β, IL-6, TNF-α, CCL2, CCL5, CCL7, and CXCL10) were measured by Q-PCR using the CFX96 real-time PCR system (Bio-Rad). PCR cycling conditions were as follows: 95 °C for 3 min, 45 cycles of 95 °C for 15 s, and 60 °C for 1 min. Gene expression was calculated using the formula 2^−[Ct(target gene)−Ct(β-actin)], as described previously [27]. Cytokine and chemokine levels are presented as the fold increase compared to noninfected controls or AHCC-treated samples.

### 2.6. Plaque Assay

Vero E6 cells were seeded in 6-well plates and incubated at 37 °C. Lung tissue homogenates were serially diluted (10-fold) in DMEM (Gibco, Billings, MT, USA) with 2% FBS (HyClone, Logan, UT, USA), and 0.2 mL was used to infect cells at 37 °C for 1 h. After incubation, samples were overlaid with MEM (Gibco, Billings, MT, USA) with 8% FBS and 1.6% agarose (Promega, Madison, WI, USA). After 48 h, plates were stained with 0.05% neutral red (Sigma-Aldrich, St. Louis, MO, USA) and plaques were counted to calculate virus titers expressed as PFU/mL.

### 2.7. Histology

Lung tissues were fixed in 10% formalin (Thermo Fisher Scientific, Waltham, MA, USA) for three days before embedment in an optimal cutting temperature compound. H&E staining was performed at the Histopathology Laboratory Core of UTMB.

### 2.8. IgM and IgG ELISA

Plates were coated with 100 ng/well of SARS-CoV-2 RBD protein (RayBiotech, Norcross, GA, USA) overnight at 4 °C. Plates were washed twice with PBS-T (0.05% Tween 20; Sigma-Aldrich, St. Louis, MO, USA) and blocked with PBS containing 8% FBS (HyClone, Logan, UT, USA) for 2 h at room temperature (RT). Serially diluted (4-fold, starting with 1:40) sera samples were added and incubated for 1 h at RT. This was followed by a 1 h incubation with HRP-conjugated goat anti-mouse IgM or IgG antibodies (Southern Biotech, Birmingham, AL, USA). 3,3′,5,5′ Tetramethylbenzidine (TMB, BD Biosciences, San Jose, CA, USA) was added to the well for 5 min. Then, stop solution was added (Thermo Fisher Scientific, Waltham, MA, USA), and the absorbance was read at 450 nm by a BioTek Cytation 7 plate reader. Binding endpoint titers were determined using a cutoff value determined by the mean of the negative controls (noninfected control-fed or AHCC-fed samples) + 3 x standard deviation (SD).

### 2.9. Flow Cytometry

Lung tissues were digested with 0.05% collagenase type IV (Thermo Fisher Scientific, Waltham, MA, USA) in RPMI 1640 Medium (Gibco, Billings, MT, USA) at 37 °C with 5% CO_2_ for 45 min, and a single-cell suspension was prepared by passing the lung homogenates through a 70 μm cell strainer followed by red blood cell lysis. Splenocytes or lung leukocytes were stained with antibodies for CD3 and TCRγδ (e-Biosciences, San Diego, CA, USA). After staining, the cells were fixed with 2% paraformaldehyde in PBS and examined using a C6 flow cytometer (BD Biosciences, San Jose, CA, USA). Live cells were enriched on the basis of forward and side light scatter. Data were analyzed with a CFlow Plus flow cytometer (BD Biosciences, San Jose, CA, USA).

### 2.10. Intracellular Cytokine Staining (ICS)

As described previously [28], splenocytes were incubated with SARS-CoV-2 S peptide pools (1 μg/mL, Miltenyi Biotec, Auburn, CA, USA) for 5 h in the presence of BD GolgiPlug (BD Biosciences, San Jose, CA, USA). Briefly, cells were stained with antibodies for CD4 or CD8, fixed in 2% paraformaldehyde, and permeabilized with 0.5% saponin before adding anti-IFN-*γ*, anti-TNF-α, or control rat IgG1 (e-Biosciences). Samples were processed with a C6 Flow Cytometer instrument. Dead cells were excluded based on forward and side light scatter. Data were analyzed with a CFlow Plus Flow Cytometer (BD Biosciences, San Jose, CA, USA).

### 2.11. IFN-γ ELISPOT

ELISPOT plates (Millipore Ltd., Burlington, VT, USA) were coated with anti-IFN-γ capture Ab (Cellular Technology Ltd., Shaker Heights, OH, USA) at 4 °C overnight. Splenocytes or lung leukocytes were then stimulated with SARS-CoV-2 S peptide pools (2 μg/mL, Miltenyi Biotec, Auburn, CA, USA) for 24 h at 37 °C. Cells stimulated with anti-CD3 (1 μg/mL, e-Biosciences, San Diego, CA, USA) or medium alone were used as controls. Next, cells were incubated with biotin-conjugated anti-IFN-γ (Cellular Technology Ltd., Shaker Heights, OH, USA) for 2 h at room temperature, and then alkaline phosphatase-conjugated streptavidin (Cellular Technology Ltd., Shaker Heights, OH, USA) for 30 min. After washing, plates were scanned using an ImmunoSpot 6.0 analyzer and analyzed by ImmunoSpot software to determine the spot-forming cells (SFC) per 10^6^ splenocytes or leukocytes.

### 2.12. Statistical Analysis

A survival curve comparison was performed using GraphPad Prism software 9.4.1, which uses the log-rank test. Values for viral load, cytokine production, antibody titers, and T cell response experiments were compared using Prism software statistical analysis and are presented as means ± SEM. The *p* values of these experiments were calculated with a nonpaired Student’s *t* test.

## 3. Results

### 3.1. Oral Uptake of AHCC Enhances Host Resistance to SARS-CoV-2 Infection in K18-hACE2 and BALB/c Mice

**Figure 1:**
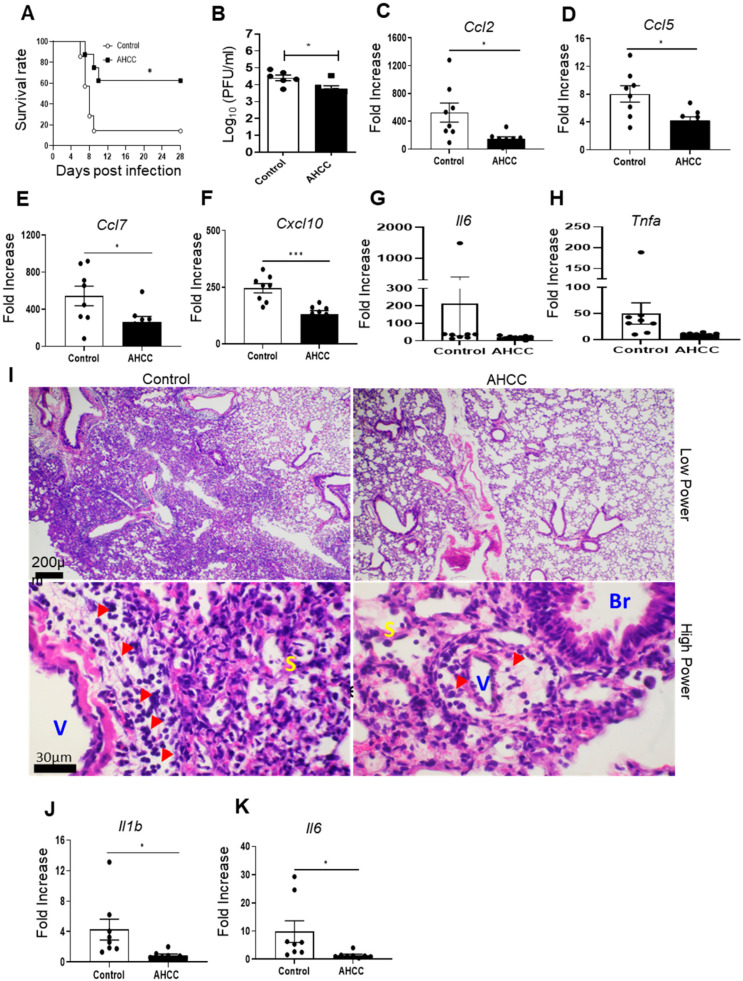
AHCC supplementation increases host survival following SARS-CoV-2 infection in K18-hACE2 mice. Six-week-old K18-hACE2 mice were orally fed with AHCC or water (control) every other day **five** times prior to infection and once on day 1 post virus challenge with the SARS-CoV-2 prototype strain. (**A**) Survival rate; AHCC-fed (n = 8); Control (n = 7), (**B**–**H**) Lung viral load and chemokine and cytokines levels at day 6 post infection determined by plaque assay or Q-PCR. Data are presented as the fold increase compared to the noninfected control or AHCC-treated samples. (**I**) Representative images show inflammation in the control group (top left panel) and AHCC-fed group (top right panel). At a high power view, there are more perivascular/peribronchiolar infiltrations with lymphocytes and monocytes/macrophages in the control group (lower left panel) compared to the AHCC-fed group (lower right panel). V, artery/arteriole; Br, bronchiole; S, septum. Bar = 200 µm in top panels, Bar = 30 µm in lower panels. (**J**,**K**) Brain cytokine levels at day 6 post infection determined by Q-PCR. Data are presented as the fold increase compared to noninfected control or AHCC-treated samples. Data are presented as means ± standard error of the mean (s.e.m). *** *p* < 0.001, or * *p* < 0.05 compared to the control group.

To determine the effects of AHCC supplementation on the host response to COVID-19 infection, we used two mouse models of SARS-CoV-2 infection. Initially, K18-hACE2 mice were orally fed with AHCC or water (control) every other day five times prior to infection and once on day 1 post the virus challenge. Mice were intranasally (i.n.) challenged with 5 × 10^3^ PFU of the SARS-CoV-2 USA-WA1/2020 prototype strain and monitored daily for morbidity and mortality. K18-hACE2 mice fed with AHCC showed a higher survival rate (62%) compared with the infected control group (14%) within a 4-week interval (Figure 1A, *p* < 0.05). SARS-CoV-2-infected K18-hACE2 mice started to display severe clinical symptoms, including encephalitis and mortality at around day 6 post infection (data not shown). Thus, to further understand viral pathogenesis, lung tissues were collected to determine viral loads and tissue inflammation and pathology at day 6. There was a 16% reduction in the viral titers of lung tissues of AHCC-fed mice compared to the control group (Figure 1B). Since the virus-induced cytokine storm in the lungs caused by proinflammatory cytokines such as IL-6, TNF-α, and IL-1β and inflammatory immune cell-induced tissue damage mediated by monocyte and lymphocyte-attracting chemokines is known to be associated with COVID-19 disease severity in humans [29,30,31], we next determined chemokine production in murine lungs by Q-PCR. We observed that AHCC treatment significantly diminished the levels of CCL2, CCL5, CCL7, and CXCL10 in the lungs of the treated group compared to the control group by an average of more than 50% (Figure 1C–F). There was a trend of lower levels of TNF-α and IL-6 in the AHCC-fed group (Figure 1G,H). Similarly, the lung pathology examination revealed about 20% and 80% inflammation in AHCC-fed and the control groups, respectively, with significantly more perivascular and peribronchiolar infiltrations, including lymphocytes and monocytes/macrophages, detected in the lungs of the control group. The infiltration expanded into the septum as the infected control group showed thicker septa than that of the AHCC-fed group (Figure 1I, top and low panels). SARS-CoV-2 disseminated into the brain and induced neuroinvasive diseases and death in K18-hACE2 mice [10,11]. We noted that, at day 6 post SARS-CoV-2 infection, AHCC administration caused a trend of lower viral loads in the brain (data not shown) but significantly reduced the levels of proinflammatory cytokines, such as IL-1β and IL-6, which are associated with neuroinflammation compared to the control group (Figure 1J,K).

**Figure 2:**
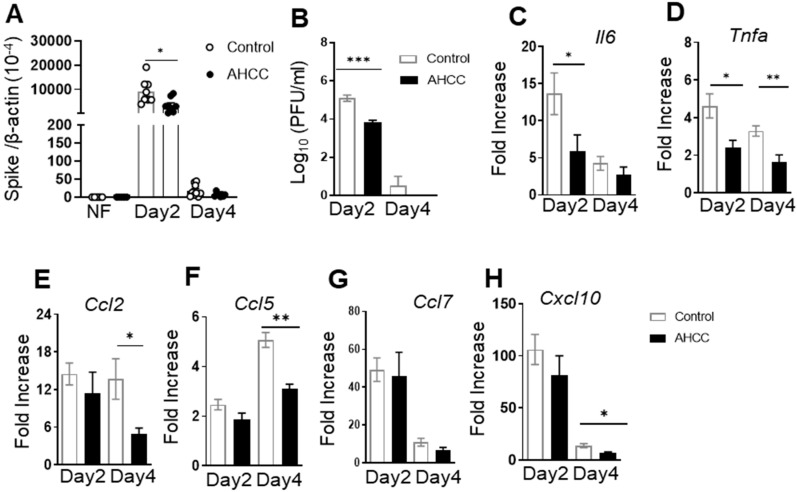
AHCC supplementation increases host resistance to SARS-CoV-2 infection in BALB/c mice. Six-week-old BALB/c mice were fed with AHCC or water (controls) every other day five times prior to infection, and on day 1 post challenge with the mouse-adaptive SARS-CoV-2 strain CMA4. (**A**,**B**) Lung viral load at days 2 and 4 post infection by Q-PCR (**A**) and plaque assays (**B**). Noninfected (NF) samples were collected following feeding five times with water or AHCC. (**C**–**H**) Lung cytokine/chemokine levels at days 2 and 4 post infection determined by Q-PCR. Data are presented as the fold increase compared to mock-infected control or AHCC-treated samples. Data are presented as the means ± s.e.m. n = 8 per group for noninfected mice. n = 6 and n = 5 for the infected control group and AHCC-treated group respectively. *** *p* < 0.001, ** *p* < 0.01, or * *p* < 0.05 compared to control group.

We next performed AHCC treatment in immunocompetent mice. Young adult BALB/c mice were fed with AHCC or water (controls) every other day five times prior to infection and on day 1 post challenge. Mice were then infected i.n. with 10^4^ PFU mouse-adapted SARS-CoV-2 strain CMA4. The mouse-adapted strain infects the lungs and causes mild inflammation with infection peaking around day 2 [14]. Thus, mice were euthanized on day 2 and day 4 post infection to measure viral loads and lung inflammation by Q-PCR and plaque assays. As shown in Figure 2A,B, there was about 60% less viral RNA detected in the lung tissues of AHCC-fed mice compared to the control group at day 2 (*p* < 0.05). Plaque assays showed that viral titers in the lung decreased by 15-fold (*p* < 0.001) in the AHCC-fed group. At day 4 post infection, the viral loads in both groups were either reduced or nondetectable, and the differences between the two groups became nonsignificant. Both IL-6 and TNF-α levels were diminished in the lungs of the AHCC-fed group compared to that of the control groups at day 2, and this reduction extended to day 4 for the TNF-α levels (Figure 2C,D, *p* < 0.05 or *p* < 0.01). The concentrations of chemokines, including CCL2, CCL5, and CXCL10, were all markedly reduced in the lungs of the AHCC-fed group at day 4 post infection (Figure 2E–G, *p* < 0.05 or *p* < 0.01). Overall, these results suggest that AHCC oral supplementation enhances host resistance to SARS-CoV-2 infection in both mouse models.

### 3.2. AHCC Supplementation Promotes Antiviral Innate and Adaptive T Cell Responses in Both Mouse Models following SARS-CoV-2 Infection and Increases IgG Titers in BALB/c Mice

**Figure 3:**
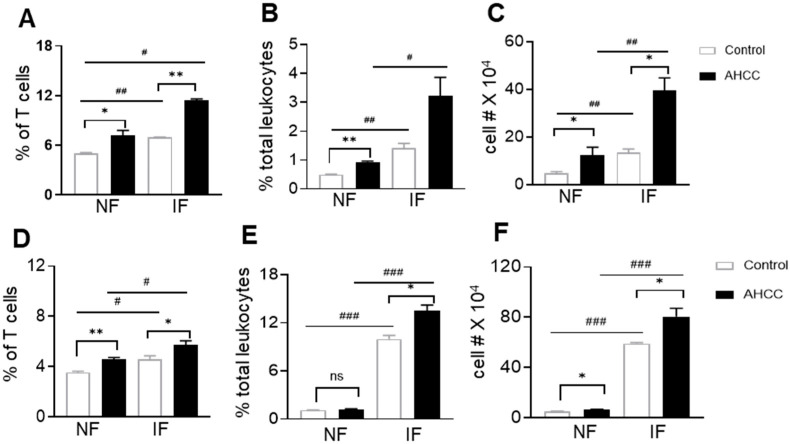
Oral administration of AHCC enhances γδ T cell expansion in the periphery prior to and after SARS-CoV-2 infection. Six-week-old BALB/c or K18-hACE2 mice were fed with AHCC or water (controls) every other day five times prior to infection, and on day 1 post challenge with the mouse-adapted SARS-CoV-2 strain CMA4 or the SARS-CoV-2 prototype strain. Splenocytes and lung leukocytes were isolated before or at day 4 post infection and stained with antibodies for CD3 and TCRγδ. (**A**–**C**): BALB/c mice. (**D**–**F**): K18-hACE2 mice. (**A**,**D**): Percent positive of γδ T cells among splenic T cells. (**B**,**C**,**E**,**F**): Percent of γδ T cells (**B**,**E**) and total number (**C**,**F**) among lung leukocytes, respectively. Noninfected (NF) samples were collected following feeding five times with water or AHCC. IF: infected samples. Data are presented as the means ± s.e.m. and are representative of 2 similar experiments (n = 3 for noninfected groups and n = 2 for infected groups). ** *p* < 0.01, or * *p* < 0.05 compared to the control group. ^#^ *p* < 0.01,^##^ *p* < 0.01, or ^###^ *p* < 0.001 compared to the noninfected group.

To understand AHCC-mediated protection in host immunity, we next measured innate and adaptive immune responses in SARS-CoV-2 infected immunocompetent mice. γδ T cells, which constitute about 5% of cells in the lymphatic organs and epithelia of nonlymphatic organs in mice and humans, are known to display antiviral activity [32,33]. We first examined γδ T cell expansion in the periphery of AHCC-treated BALB/c mice infected with SARS-CoV-2. In the spleen, γδ T cell expansion was observed in both control and AHCC-treated mice following infection. AHCC supplementation enhanced the percentage of γδ T cells both before and after SARS-CoV-2 infection compared with the control group (Figure 3A). In the lungs, γδ T cell expansion was also observed in control and AHCC-treated mice following infection. γδ T cell expansion was enhanced in terms of the percentage of lung leukocytes and the cell number compared to the control group, both prior to infection and at day 4 post infection (Figure 3B,C). We observed similar effects of AHCC-treatment on γδ T cell expansion in the spleens (Figure 3D) and lungs (Figure 3E,F) of K18-hACE2 mice prior to and after infection, except no difference in the percentage of γδ T cells in the lungs was observed between the control and AHCC-treated groups prior to infection (Figure 3E). Overall, these results suggest that AHCC supplementation promotes γδ T cell expansion in both lymphatic and nonlymphatic organs before and after SARS-CoV-2 infection.

**Figure 4:**
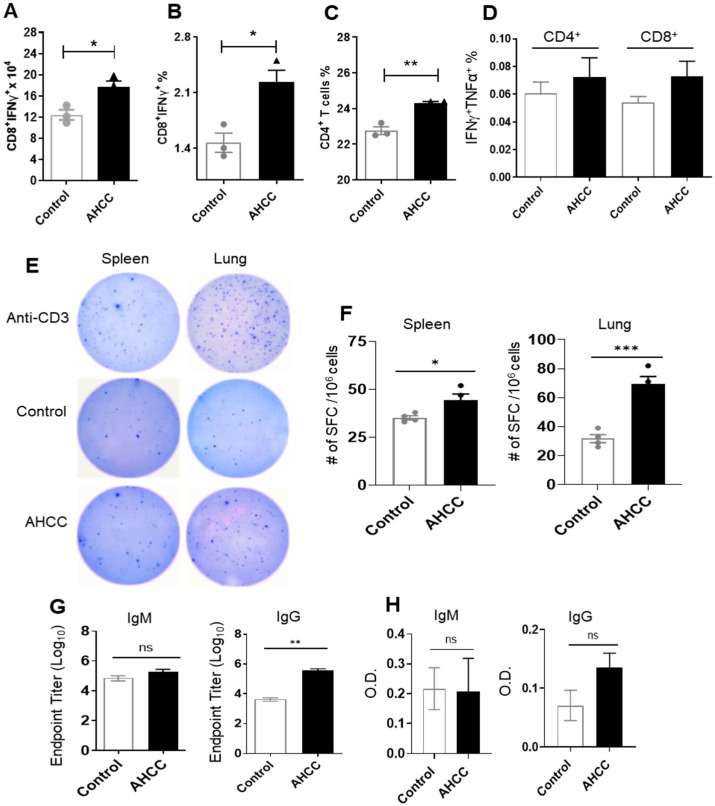
AHCC treatment promotes SARS-CoV-2-specific T and humoral immune responses. Six-week-old BALB/c mice (**A**–**D**,**G**) or K18 hACE2 mice (**E**,**F**,**H**) were fed with AHCC or water (controls) every other day five times prior to infection and on day 1 post challenge with the mouse-adapted SARS-CoV-2 strain CMA4. (**A**–**D**): At day 4, splenocytes were cultured ex vivo with SARS-CoV-2 S peptide pools for 5 h and stained for IFN-γ, TNF-α, CD3, and CD4 or CD8. The total number of CD8^+^ IFN-γ^+^ (A) or the percent of IFN-γ^+^ CD8^+^ (**B**) or IFN-γ^+^ CD4^+^ (**C**) cell subsets and the percent of IFN-γ^+^TNFα^+^ (**D**) of CD8^+^ and CD4^+^ cell subsets are shown. n = 3 for Panels (**A**–**D**). (**E**,**F**) ELISPOT assay of SARS-CoV-2 -specific lung and splenic T cells. Lung leukocytes and splenocytes were stimulated with SARS-CoV-2 S peptides for 24 h. Spot forming cells (SFC) were measured by IFN-γ ELISPOT. N = 4. (**G**,**H**) Serum IgM and IgG titers. (**G**) Sera endpoint IgG, IgM titers against SARS-CoV-2 RBD at day 8. (**H**) O.D. values by ELISA. at day 4 post infection were determined by indirect ELISA. n = 9. Data are presented as the means ± s.e.m. *** *p* < 0.001, ** *p* < 0.01 or * *p* < 0.05 compared to control group.

Both humoral and T cell-mediated adaptive immune responses are critical for virus clearance and disease control of coronavirus infection [34,35]. At day 4 post infection, we assessed systemic T cell responses. Splenocytes were isolated from both control and AHCC-fed BALB/c mice and were then treated ex vivo with peptide pools of the SARS-CoV-2 S protein. The number and percentage of IFNγ-producing effector CD8^+^ T cells in AHCC-fed mice were 40–50% higher than those of the control group (Figure 4A,B). AHCC also triggered a modest increase (7%) in the percentage of splenic CD4^+^ T cells (Figure 4C). Although not significant, we observed a biological trend of enhanced IFNγ^+^TNFα^+^ of CD4^+^ and CD8^+^ T cells in the AHCC-fed group (Figure 4D). To assess the effect of AHCC treatment on SARS-CoV-2 specific effector T cell functions in K18-hACE2 mice, at day 4 post infection, splenocytes and lung leukocytes were collected from control and AHCC-fed mice and stimulated with S peptide pools. Splenocytes and lung leukocytes of AHCC-fed K18 HACE2 mice produced 25% and 124% higher levels of IFNγ than the control group, respectively (Figure 4E,F). To assess SARS-CoV-2-specific antibody responses, we noted that S-specific IgG titers were 85-fold higher in the AHCC-fed BALB/c mice at day 8 post infection. However, no differences were detected on IgM titers in these mice and IgG and IgM titers in K18-hACE2 mice between the two groups (Figure 4G,H). Overall, these results suggest that AHCC induces SARS-CoV-2-specific T helper (Th)-1 cell prone adaptive immune responses in both mouse models and increases the IgG response in BALB/c mice.

## 4. Discussion

COVID19 infection induces various clinical manifestations, ranging from mild symptoms to severe pneumonia, critical multiorgan failure, and death. K18 hACE2 mice are highly susceptible to SARS-CoV-2 infection and display weight loss, interstitial pneumonitis, encephalitis, and lethality. A mouse-adapted strain of SARS-CoV-2 replicates in the lung of young adult immunocompetent mice and causes mild clinical symptoms [10,11,14]. In this study, we demonstrated that oral supplementation with AHCC prior to and after SARS-CoV-2 infection reduces viral replication and attenuates virus-induced inflammation in the lung tissues in both mouse models. AHCC treatment also significantly reduced SARS-CoV-2-induced mortality in K18 hACE2 mice. These results suggest that AHCC supplementation protects mice from severe and mild COVID-19 infection.

γδ T cells lack major histocompatibility complex (MHC) restriction, giving them the potential to respond to antigens without the requirement for conventional antigen processing. They can rapidly proliferate after parasitic, bacterial, and viral infections and produce inflammatory cytokines, such as IFN-γ and TNF-α. Thus, these cells are considered the first-line of host defense against microbial infection [36]. The antiviral activities of γδ T cells have been well documented in humans and animals [32,37]. Lymphopenia, particularly the reduction of γδ T cells, have been reported to contribute to the disease progression in COVID-19 patients [33,38]. Here, AHCC supplementation increases γδ T cell number before and after SARS-CoV-2 infection and, thus, represents a potential new treatment option to reduce the COVID-induced disease severity.

Reductions in the CD4^+^ and CD8^+^ αβ T cell numbers and decreased IFN-γ production by αβ T cells have also been linked to the disease severity observed in COVID-19 patients [35,39]. In this study, we demonstrated that AHCC increases Th1 prone immune responses following SARS-CoV-2 infection, which provides additional insight into AHCC-mediated protective immunity. Although some reports suggest that greater antibody responses are associated with higher viral loads in nasopharyngeal swabs from severely ill COVID-19 patients [40,41], multiple lines of evidence suggest that the development of early humoral immune responses targeting the S protein, in particular the S1 or RBD domains, lead to decreased viral loads and ultimate recovery from COVID-19 [42,43]. Here, we noted an increase in RBD-specific IgG titers in AHCC-fed BALB/c mice. Nevertheless, this effect was observed on neither IgM titers of the same mice, nor in AHCC-treated K18-hACE2 mice. Overall, it appears that Th1-prone T cell responses are critical for AHCC-mediated host protection in both mouse models.

AHCC has been used as a dietary supplement to enhance the immune system and has been shown to be an immune modulator in the enhancement of host defense against microbial infections [18] as well as against tumor malignancy [17,44] in animal models. Although the underlying mechanisms of its immunomodulatory effects are still under investigation, its high content of α-1,4-glucans has been proposed as the major element contributing to its positive effects on the immune systems of humans and rodents [17,45,46]. α-1,4-glucans exert immunostimulatory effects and induce immune receptors, such as toll-like receptor (TLR)s, which are expressed on many cell types [47,48]. Previous studies have shown that AHCC has immunostimulatory effects on intestinal epithelial cells and monocytes via the activation of TLR2 and TLR4 signaling pathways [49,50]. γδ T cells express multiple TLRs, including TLR 2, 3, and 4. The activation of TLR signaling modulates the function of γδ T cells [51,52]. α-1,4-glucans can also directly trigger cytokine production by αβ T cells via the activation of TLR signaling pathways. Alternatively, α-1,4-glucan-enriched AHCC triggers TLR-expressing antigen presenting cells, including macrophages and dendritic cells, which then promote T cell priming and further differentiation into Th1-prone cells [47,53]. Thus, these previous reports suggest that AHCC modulates innate and adaptive T cell responses by the direct or indirect induction of innate signaling pathways on innate immune cells via highly enriched α-1,4-glucans.

The COVID-19 pandemic has posed a serious threat to human health and public safety, and the development of alternative solutions, in particular, noninvasive means to decrease or attenuate COVID-19-related complications are needed. Aging, one of the known risk factors for severe COVID-19 complications, is associated with nutritional deficiencies [9]. Nutrient supplementation, including the intake of vitamin C, vitamin D, Zinc, and B3 vitamins, has been recommended to attenuate the severity of COVID-19 [54,55]. Here, we demonstrated that the oral administration of AHCC, a dietary supplement sourced from edible mushrooms, can be used as an immune enhancer to improve protective innate and adaptive immune factors and thus decrease virus infection and host susceptibility to COVID-19-induced severe diseases. Our results suggest that AHCC supplementation can serve as a novel treatment option to modulate host protective immunity and control both mild and severe cases of COVID-19 infection.

## Data Availability

The data that support the findings of this study are available from the corresponding author upon reasonable request.
